# Sex-Related Differences in the Association Between Sleep Apnea and Subsequent Urinary Incontinence Diagnosis

**DOI:** 10.3390/clockssleep7040065

**Published:** 2025-11-07

**Authors:** Lara Ilona Becker, Céline Vetter, Karel Kostev, Matthias Kalder

**Affiliations:** 1 Department of Gynecology and Obstetrics, University Hospital, Philipps-University Marburg, Baldingerstraße, 35043 Marburg, Germany; 2 IQVIA, Real World Solutions, 60549 Frankfurt am Main, Germany

**Keywords:** sleep apnea, urinary incontinence, lower urinary tract symptoms, Germany, cohort study

## Abstract

Objective: An association between sleep apnea and various urological symptoms has been reported in the literature. Therefore, the aim of this study is to analyze sex-related differences in the association between sleep apnea und subsequent urinary incontinence diagnosis. Methods: This study examined the incidence of urinary incontinence in a matched pair cohort with and without sleep apnea treated in 1293 general practices in Germany between January 2005 and December 2022 (74,453 vs. 372,256 individuals). The five-year cumulative incidence of urinary incontinence in the cohorts with and without sleep apnea was studied using Kaplan–Meier curves and log-rank tests. Finally, a univariable Cox regression analysis was conducted to assess the association between sleep apnea and urinary incontinence. Stratified analyses were conducted by sex (male/female) and age group (18–50 years, 51–60 years, 61–70 years, >70 years). Results: Sleep apnea was significantly associated with urinary incontinence as compared to individuals without sleep disorder diagnosis (5.1% vs. 4.3%; *p* < 0.001), and this association remained robust in females (HR: 1.38; 95% CI: 1.29–1.46), but not in males (HR: 1.02; 95% CI: 0.96–1.08) In females, the association was strongest in the age group 51–60 years (HR: 1.98; 95% CI: 1.71–2.30). Conclusions: In conclusion, this study reports a significant association between sleep apnea and subsequent urinary incontinence diagnosis. Sex- and age-related differences should be taken into account, as associations were stronger for middle-aged females followed by younger females and no significant association was found regarding males.

## 1. Introduction

Obstructive sleep apnea (OSA) is a sleep-related breathing disorder caused by repetitive collapse of the upper airway during sleep, resulting in a partial reduction in airflow (hypopnea) or a complete cessation of airflow (apnea). These episodes lead to intermittent hypoxemia and sleep fragmentation [1,2]. Sleep apnea is typically associated with daytime sleepiness but is also linked to multiple co-diagnoses including cardiovascular comorbidities, metabolic dysfunction, and lower urinary tract symptoms [2,3]. The incidence of sleep apnea varies between 2% and 14% in the general population, increasing markedly with advancing age [1,4].

Associations have been identified between sleep apnea and various urological symptoms such as sexual function, nocturia [5,6], overactive bladder, and urinary incontinence [7,8].

Previous studies have primarily investigated nocturia in relation to sleep apnea [5,6] while data on urinary incontinence remain limited. Nocturia can be a symptom of an overactive bladder (OAB), although daytime urinary urgency and facultative, urinary incontinence are more typical symptoms of the condition [9]. Patients with OAB describe a higher frequency of urination, also during the night [8]. Up to 15% of males and 40% of females are affected by OAB and, correspondingly, urinary incontinence, which is characterized by unintentional urine leakage [9]. Interestingly, the prevalence of OAB in patients diagnosed with sleep apnea is distinctly higher than among the overall population [10]. Previous studies have even observed an association between the severity of sleep apnea and OAB [7,10,11]. Females suffering from sleep apnea are particularly likely to report symptoms consistent with OAB [9,12]. Urinary incontinence has also been demonstrated to be more common in female patients [8].

The association between sleep apnea and urological symptoms such as incontinence can have a significant impact on quality of life, with implications for social life and mental and sexual health [9,10]. Unfortunately, there are still just a limited number of studies addressing the prevalence of OAB symptoms besides nocturia in patients diagnosed with sleep apnea, and the mechanisms at play between OAB, its respective symptoms, and sleep apnea are not fully understood [9,11,12]. As males are diagnosed with sleep apnea more often in general [4], there is a lack of data concerning women with both sleep apnea and urological symptoms such as incontinence. Therefore, the aim of this study is to analyze sex-related differences in the association between sleep apnea und subsequent urinary incontinence diagnosis. We hypothesize that sleep apnea is significantly associated with a subsequent diagnosis of urinary incontinence, and that this association is stronger in women than in men.

## 2. Results

### 2.1. Basic Characteristics of the Study Sample

The present study included 74,453 individuals with sleep apnea and 372,256 individuals without sleep disorders. The basic characteristics of the study patients are displayed in Table 1. The mean age was 58 (standard deviation (SD): 1 year), and 30% were women. Patients visited their GPs an average of 8 times per year during the follow-up period. Due to the matched pairs design, no significant differences were observable between both cohorts in terms of age, sex, visit frequency, and CCI (Table 1).

### 2.2. Association Between Sleep Apnea and Subsequent Urinary Incontinence Diagnosis

**Figure 1:**
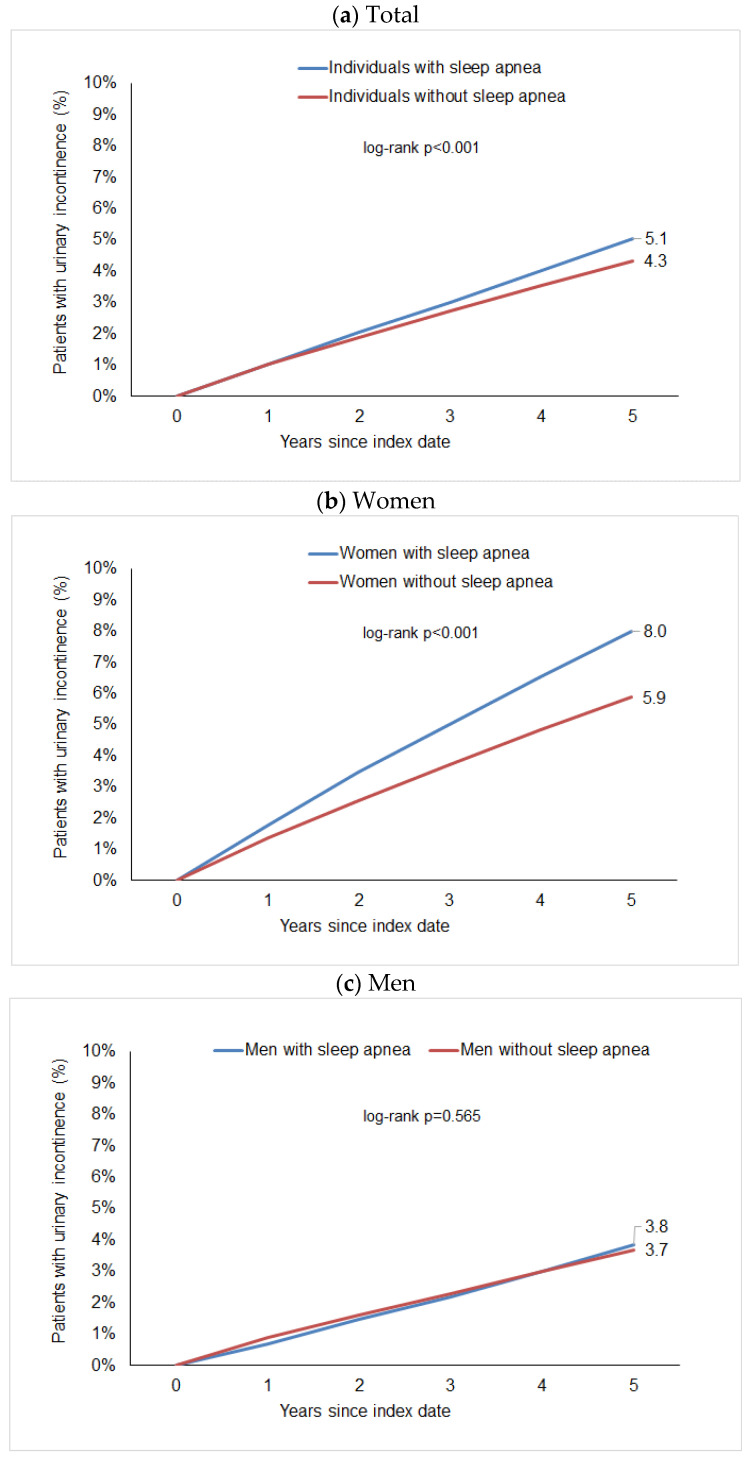
Cumulative incidence of urinary incontinence in individuals with and without sleep apnea.

After up to five years of follow-up, 5.1% of individuals with sleep apnea and 4.3% of individuals without sleep disorders were diagnosed with urinary incontinence (Figure 1a). These proportions were 8.0% vs. 5.9% in females (Figure 1b) and 3.8% vs. 3.7% in males (Figure 1c).

The regression analysis showed a significant but slight association between sleep apnea and subsequent urinary incontinence diagnosis (HR: 1.15; 95% CI: 1.11–1.20). Post hoc power calculations show a power greater than 0.99 for this association. However, we observed a considerable difference between women and men. In women, the Hazard Ratio for the association between sleep apnea and subsequent urinary incontinence was 1.38 (95% CI: 1.29–1.46) and was strongest in the age group 51–60 years (HR: 1.98; 95% CI: 1.71–2.30) followed by 18–50 years (HR: 1.68; 95% CI: 1.34–2.11), but was weaker and not significant in women aged > 70 years (HR 1.10; 95% CI: 1.00–1.20). No significant association was observed between sleep apnea and subsequent urinary incontinence diagnosis in males (HR: 1.02; 95% CI: 0.96–1.08) (Table 2).

## 3. Discussion

The aim of this study was to examine the association between sleep apnea and the 5-year incidence of urinary incontinence in 74,453 patients diagnosed with sleep apnea and 372,265 individuals without sleep apnea in terms of sex-related differences. The study shows a significant association between sleep apnea and subsequent urinary incontinence diagnosis: 5.1% of individuals with sleep apnea and 4.3% of individuals without sleep disorders were diagnosed with urinary incontinence (HR: 1.15; 95% CI: 1.11–1.20). Looking at the data for males and females separately, there is a significant association between sleep apnea and subsequent urinary incontinence diagnosis in women (HR: 1.38; 95% CI: 1.29–1.46) but not in men (HR: 1.02; 95% CI: 0.96–1.08). In addition, the association between sleep apnea and subsequent urinary incontinence in females differed by age, with stronger associations observed for females between 51 and 60 years (HR: 1.98; 95% CI: 1.71–2.30) followed by those aged 18 to 50 years (HR: 1.68; 95% CI: 1.34–2.11) and no significant results in elderly females aged over 70 years (HR 1.10; 95% CI: 1.00–1.20).

While previous studies have often focused on nocturia as a key lower urinary tract symptom associated with sleep apnea, the present study specifically addresses urinary incontinence. Although nocturia and urinary incontinence may share underlying mechanisms, such as increased nocturnal urine production and detrusor overactivity, our results demonstrate that urinary incontinence itself warrants separate consideration.

As data focusing explicitly on urinary incontinence is rare, the current findings also have to be interpreted against the background of related urological symptoms. Most data in this area are related to sleep apnea and nocturia. Some studies linked sleep apnea with the symptom complex of overactive bladder, which can be accompanied by urinary incontinence as well as nocturia [9]. After discussing the association between sleep apnea and subsequent urinary incontinence diagnosis as well as its general pathophysiology, this section will focus on sex-related differences before addressing age-related differences.

Sleep apnea and lower urinary tract symptoms such as urinary incontinence share several risk factors. For example, both are associated with diabetes and obesity [13,14]. In women, the relationship between obesity and urinary incontinence may be partly influenced by parity, as multiple pregnancies can contribute to both higher body weight and pelvic floor dysfunction. Beyond these shared risk factors, several physiological pathways may link sleep apnea directly to urinary incontinence. Repetitive upper airway obstruction in sleep apnea causes intermittent hypoxia, sympathetic activation, and oxidative stress, which can contribute to detrusor overactivity and pelvic floor muscle dysfunction [1,2,8,9,11]. Increased intra-abdominal pressure during nocturnal respiratory effort may further strain pelvic floor structures, while sleep fragmentation and frequent arousals could impair cortical control of micturition and exacerbate bladder instability [6]. These mechanisms may jointly explain why urinary incontinence occurs more frequently among individuals with sleep apnea, particularly in females.

Previous research has also emphasized the role of intermittent hypoxia in the pathophysiology of both sleep apnea and urinary incontinence. Hypoxia-related oxidative stress and inflammation can cause endothelial dysfunction, peripheral nerve injury, and increased sympathetic activity, which in turn may promote detrusor instability and spontaneous bladder contractions [8,9,10,12]. These mechanisms provide further support for a physiological link between sleep apnea and urinary incontinence.

Disturbances in circadian water homeostasis may also play a role in the association between sleep apnea and urinary incontinence. Negative intrathoracic pressure during airway obstruction can lead to elevated atrial natriuretic peptide and reduced antidiuretic hormone secretion, resulting in increased nocturnal urine production [5,6,9]. Although this mechanism primarily explains nocturia, it may secondarily exacerbate urge or stress incontinence in susceptible individuals. Central nervous system dysregulation, including altered hypothalamic activity and sleep fragmentation, may further impair voiding control [15]. Moreover, previous studies have shown that women with urge urinary incontinence experience greater sleep disturbance than continent women [16].

In general, previous studies focused on the association between sleep apnea and nocturia, general lower urinary tract symptoms and the overactive bladder symptom complex. The latter may include nocturia as well as urinary incontinence [9]. Unfortunately, most previous studies did not focus explicitly on urinary incontinence as addressed in the present study but instead described the association between sleep apnea and OAB in general. As data focusing on sleep apnea and urinary incontinence are rare, studies examining the wide-ranging symptom complex of OAB must be used to discuss the present findings.

The present study shows a significant association between sleep apnea and subsequent urinary incontinence diagnosis. In line with this finding, the prevalence of OAB in patients diagnosed with sleep apnea is distinctly higher than in the total population [10]. Up to 50% of patients with sleep apnea in a mixed-sex and up to 80% in a completely female group also suffer from OAB [11,12]. Furthermore, there may be an association between the severity of sleep apnea and the number of OAB symptoms. Kemmer et al. showed that males with moderate and severe sleep apnea present higher incidences of OAB symptoms than those with mild sleep apnea [9]. Correspondingly, a review by Clerget at al. described a correlation between the severity of OAB and the severity of sleep apnea, especially when the latter is moderate or severe [17]. In addition, data indicate a positive association between the severity of sleep apnea and the severity of nocturia as well as the severity of daytime urinary frequency [9]. By contrast, Ipekci et al. found no statistically significant connection between the severity of sleep apnea and the prevalence of OAB in females [11]. Similarly, Yilmaz et al. found no significant difference between the severity of sleep apnea and the prevalence of OAB in a mixed-sex study population [8].

Focusing on urinary incontinence as the subject of this study, several cross-sectional studies among males have demonstrated that patients with sleep apnea are more likely to suffer from urinary incontinence than men without sleep apnea [18]. These studies have also shown an association between the severity of urinary incontinence and the severity of sleep apnea [9]. Conversely, Truncer et al. observed no significant difference between the severity of sleep apnea with respect to the incidence of OAB symptoms such as urgency urinary incontinence in males [19]. Furthermore, no higher prevalence of OAB and urinary incontinence has been found in young males [19].

Looking at females, Myer et al. observed a high prevalence of apnea among urogynecology patients: Female participants who screened as high-risk for obstructive sleep apnea were more likely to report disturbing bladder symptoms and suffer from more severe incontinence [20]. Ipekci et al. demonstrated that around one third of females with sleep apnea with or without OAB suffer from urinary incontinence [11]. For a mixed-sex group, not even one in five people with sleep apnea also suffered from urinary incontinence [12]. In conclusion, no consistent association between apnea and nocturia, OAB, and urinary incontinence has been reported in previous studies. There is a need for further studies including both males and females as well as studies distinguishing clearly between urinary incontinence and related urological symptoms.

In this study, we observed a significant difference between women and men regarding the association between sleep apnea and subsequent urinary incontinence, identifying a significant association for women but not for men. Kemmer et al. showed that 39% of males suffering from sleep apnea also report symptoms consistent with OAB [9]. The association was stronger in mixed or all-female groups [11,12] and around one third of sleep apnea patients with or without OAB suffered from urinary incontinence [11,12]. Yilmaz et al. observed a significant correlation between the symptoms of OAB and urinary incontinence in female apnea patients [8]. By contrast, Zhou et al. demonstrated a significant association regarding the risk of nocturia in men but not in women [21]. Umlauf et al. found no difference between men and women [6] and Bostan et al. described a higher prevalence of nocturia in female patients with sleep apnea than in men [22]. In conclusion, studies with a distinct focus on sex-related differences are rare and inconsistent.

Several factors may explain the stronger association between sleep apnea and urinary incontinence observed in females. Anatomically, women have a shorter urethra and lower urethral closure pressure, which increases vulnerability to urinary leakage during episodes of elevated intra-abdominal pressure caused by obstructed breathing efforts in sleep apnea. Hormonal influences also play an important role: estrogen deficiency after menopause can lead to atrophic changes in the urethral mucosa and pelvic floor musculature, reducing continence control [8,14]. In addition, pregnancy and vaginal deliveries can cause pelvic floor and connective tissue damage, which may be aggravated by chronic straining or intermittent hypoxia associated with sleep apnea [11]. By contrast, urinary incontinence in males is often related to prostatic pathology, which may explain why the association between sleep apnea and urinary incontinence was not significant in men in our cohort. These sex-specific anatomical and hormonal factors likely contribute to the stronger relationship observed in females.

Age-related differences in the association between sleep apnea and subsequent urinary incontinence diagnosis in females are an incidental finding of this study and will be discussed below. The association between sleep apnea and subsequent urinary incontinence was stronger in the age group 51–60 years, followed by the group 18–50 years. No significant association was discovered in women aged over 70 years. Correspondingly, Miyauchi et al. observed that night-time frequency was significantly associated with the severity of sleep apnea in younger patients but not in elderly patients aged 65+ years [3]. Accordingly, previous studies demonstrated associations between nocturia and sleep apnea in younger but not elderly males [23,24]. Interestingly, both the prevalence of sleep apnea and urological symptoms such as nocturia or urinary incontinence increase with age [1,4,19,20]. This supports the idea that sleep apnea may have more influence on urological symptoms such as nocturnal urine frequency in younger patients. The association between sleep apnea and the development of urological symptoms may be reduced in an elderly population and may be a result of a multifactorial genesis—age-related structural changes in urinary organs may be more relevant, for example [3,5,24].

An important aspect not captured in the present analysis is the potential effect of sleep apnea treatment on urinary symptoms. Continuous positive airway pressure (CPAP) therapy is the standard treatment for obstructive sleep apnea and effectively reduces nocturnal hypoxia, arousals, and negative intrathoracic pressure. Several studies have reported improvements in overactive bladder symptoms and nocturia following CPAP treatment [10,11,12], suggesting that at least part of the pathophysiological link between sleep apnea and lower urinary tract symptoms may be reversible when nocturnal breathing is normalized. It is conceivable that similar mechanisms may apply to urinary incontinence, particularly if detrusor overactivity or pelvic floor strain are driven by chronic respiratory effort and intermittent hypoxia. However, the absence of treatment data in our study precludes assessment of this hypothesis. Future studies including therapy-related information and objective measures of sleep apnea severity could provide valuable insight into whether treatment can mitigate or reverse urinary incontinence risk.

## 4. Strengths and Limitations

This retrospective study boasts a number of strengths: the large sample size, the follow-up period of up to five years, the diagnosis of sleep apnea by licensed physicians, and the control of possible confounders such as age, sex, and a wide range of comorbidities. Nevertheless, the present study is also subject to a number of limitations that have to be mentioned.

As this was a retrospective observational study, causal relationships cannot be inferred, and residual confounding cannot be ruled out. Although we adjusted for several important factors, including age, sex, visit frequency, and comorbidities through the Charlson Comorbidity Index, the analyses were limited to variables available in the database. The CCI score was assessed at baseline only, and potential changes in comorbidities or new diagnoses during the follow-up period were not accounted for. These aspects may have influenced the observed associations.

The inclusion of patients based on ICD codes means that the possible effects of incorrect coding or updated ICD categorizations during data generation cannot be ruled out. Because diagnoses in the Disease Analyzer database are coded using ICD-10, the specific subtype of sleep apnea (obstructive, central, or mixed) could not be distinguished. However, in clinical practice, the ICD-10 code G47.3 predominantly represents obstructive sleep apnea. Moreover, the Disease Analyzer database does not include information about diagnostic methods. Therefore, it was not possible to determine whether sleep apnea diagnoses were based on polysomnography, polygraphy, or clinical evaluation. Nonetheless, in German clinical practice, sleep apnea (ICD-10: G47.3) is usually confirmed by sleep studies conducted by specialists before documentation by general practitioners.

Furthermore, no information was collected regarding the severity of sleep apnea or urinary incontinence. A further categorization of urinary incontinence (stress vs. urge vs. mixed incontinence) and an overlap with related urological symptoms, for example nocturia or OAB, would have been informative and could have allowed for a more comprehensive interpretation of our findings in the context of existing data. The availability of data regarding the association between urinary incontinence and sleep apnea was limited. Most studies focused on related symptoms such as nocturia and did not explicitly ask about urinary incontinence. This limited the comparability of data as well. Further anamnestic information on the subjects included would also have been of interest, e.g., obesity, a history of urogynecological surgery, parity in females, or benign prostate hyperplasia in men. Finally, data were generated based on the ICD codes selected by general practitioners in Germany, which may have an influence on generalizability. The inclusion of data from hospitals and specialized physicians and data collected in broader study populations could be illuminating.

## 5. Methods

### 5.1. Database

This retrospective cohort study was based on data from the Disease Analyzer database (IQVIA), which contains anonymous data on diagnoses and prescriptions as well as basic medical and demographic data from computer systems in office-based practices [25]. The database covers approximately 3000 of these practices in Germany. The sampling method for the Disease Analyzer database uses statistics from German Medical Association to determine the panel design based on specialist group, German federal state, community size category, and physician age. It has previously been shown that the panel of practices included in the Disease Analyzer database is representative of general and specialized practices in Germany [25]. In addition, data from the database have already been used in several previous studies focusing on sleep disorders [26,27] or urinary incontinence [28].

### 5.2. Study Population

**Figure 2:**
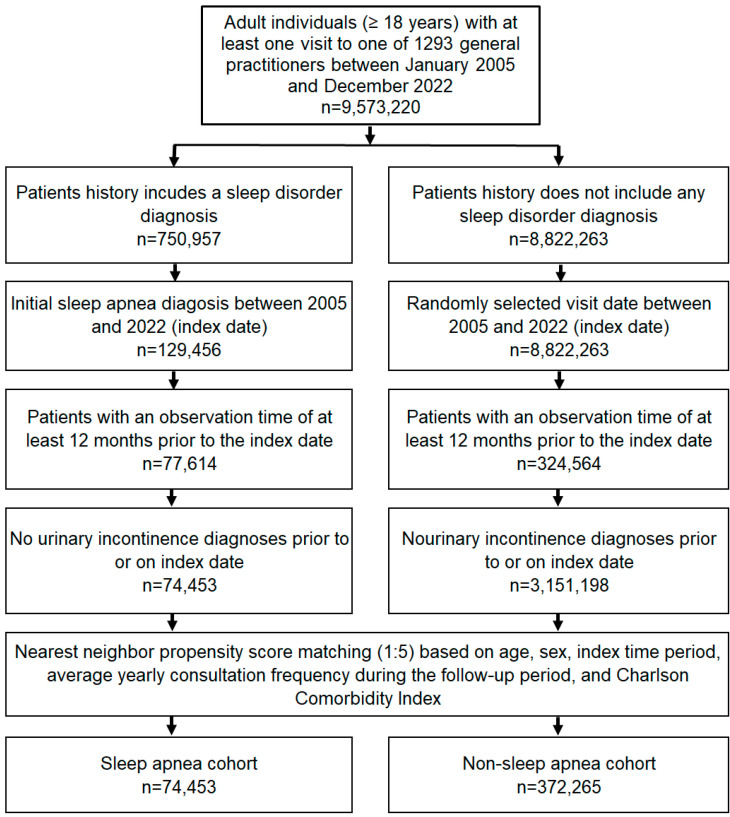
Selection of study patients.

This study included individuals aged ≥ 18 years with an initial diagnosis of sleep apnea (ICD-10: G47.3) in 1293 general practices in Germany between January 2005 and December 2022 (index date; Figure 2). Patients with diagnoses documented by GPs may also have received previous diagnoses from specialists in other practices or in hospitals. The date of initial documented sleep apnea diagnosis by a GP was considered the index date. An observation time of at least 12 months prior to the index date was a further criterion for inclusion. Patients with urinary incontinence diagnoses (ICD-10: N39.3, N39.4, R32) prior to or on the index date were excluded. After applying similar inclusion criteria, individuals without sleep disorder diagnoses (ICD-10: G47, F51) were matched to patients with sleep apnea using nearest neighbor propensity score matching (1:5) based on age, sex, index period (year), average annual number of GP consultations during the follow-up period, and Charlson Comorbidity Index (CCI). The CCI comprises a wide range of comorbidities including diabetes, cancer, cardiovascular, pulmonary, gastrointestinal, liver, renal diseases, and others [29] (Figure 2). A standardized mean difference (SMD) of less than 0.1 was allowed, indicating that adequate covariate balance between cohorts had been achieved.

### 5.3. Study Outcomes and Statistical Analyses

The main study outcome was the initial diagnosis of urinary incontinence (ICD-10: N39.3, N39.4, R32) within five years following the index date as a function of sleep apnea. In our study, we included stress urinary incontinence (ICD-10: N39.3) diagnoses as part of the broader urinary incontinence outcome because this diagnosis is clinically and epidemiologically relevant in the context of sleep apnea-related pelvic floor dysfunction, especially among women. The five-year cumulative incidence of urinary incontinence in the cohorts with and without sleep apnea was also studied using Kaplan–Meier curves, and these curves were then compared using the log-rank test. Finally, a univariable Cox regression analysis was conducted to assess the association between sleep apnea and urinary incontinence. The results of the Cox regression model were displayed as hazard ratios (HRs) and 95% confidence intervals (CIs). In addition, Cox regression analyses were conducted separately for males and females and for four age groups. Due to the multiple comparisons involved, a *p*-value of <0.01 was considered statistically significant. Analyses were carried out using SAS version 9.4 (SAS Institute, Cary, NC, USA).

## 6. Conclusions

In conclusion, this study reports a significant association between sleep apnea and subsequent urinary incontinence diagnosis. Sex- and age-related differences should be taken into account, as associations were stronger for middle-aged and younger females, while no significant association was found regarding males. Nevertheless, patients with sleep apnea should be asked explicitly about urological symptoms. Furthermore, we strongly recommend that patients diagnosed with urinary incontinence who report sleep disorders should be examined for sleep apnea.

## Figures and Tables

**Table 1:** Baseline characteristics of the study sample (after propensity score matching).

Variable	Proportion Among Individuals with Sleep Apnea (%) N = 74,453	Proportion Among Individuals Without Sleep Disorders (%) N = 372,265	SMD
Age (Mean, SD)	58.4 (13.3)	58.5 (13.5)	−0.011
Age 18–50	19,720 (26.5)	98,469 (26.5)	
Age 51–60	21,933 (29.5)	105,942 (28.5)	
Age 61–70	18,156 (24.4)	91,633 (24.6)	
Age > 70	14,644 (19.6)	76,191 (20.4)	
Female	21,995 (29.5)	110,452 (29.7)	−0.001
Male	52,458 (70.5)	261,813 (70.3)	
Number of physician visits per year during the follow-up (Mean, SD)	8.1 (4.0)	8.1 (4.0)	0.002
Charlson Comorbidity Score (Median, IQR)	2 (2)	2 (2)	0.005
CCI 0	9831 (13.1)	48,749 (13,1)	
CCI 1	16,472 (22.0)	81,910 (22.0)	
CCI 2	15,851 (21.3)	79,370 (21.3)	
CCI ≥ 3	32,299 (43.6)	162,236 (43.6)	

Proportions of patients are given in % unless otherwise indicated. SD: standard deviation; IQR: interquartile range; SMD: standardized mean difference.

**Table 2:** Association between sleep apnea and subsequent urinary incontinence in patients followed in general practices in Germany (univariable Cox regression models).

	Women		Men	
**Sub-Cohorts**	HR (95% CI)	*p*-Value	HR (95% CI)	*p*-Value
**Total**	1.38 (1.29–1.46)	<0.001	1.02 (0.96–1.08)	0.563
**Age 18–50**	1.68 (1.34–2.11)	<0.001	1.27 (1.00–1.61)	0.051
**Age 51–60**	1.98 (1.71–2.30)	<0.001	1.17 (1.01–1.35)	0.042
**Age 61–70**	1.54 (1.36–1.75)	<0.001	1.04 (0.93–1.16)	0.520
**Age > 70**	1.10 (1.00–1.20)	0.045	0.99 (0.91–1.06)	0.699

HR: hazard ratio; CI: confidence interval.

## Data Availability

Due to the proprietary nature of the Disease Analyzer database and IQVIA’s terms of data use agreement, research data cannot be shared.

## References

[1] Lv R., Liu X., Zhang Y., Dong N., Wang X., He Y., Yue H., Yin Q. (2023). Pathophysiological mechanisms and therapeutic approaches in obstructive sleep apnea syndrome. Signal Transduct. Target. Ther..

[2] Lévy P., Kohler M., McNicholas W.T., Barbé F., McEvoy R.D., Somers V.K., Lavie L., Pépin J.-L. (2015). Obstructive sleep apnoea syndrome. Nat. Rev. Dis. Primers.

[3] Miyauchi Y., Okazoe H., Tamaki M., Kakehi T., Ichikawa H., Arakawa Y., Mori Y., Koui F., Sugimoto M., Kakehi Y. (2020). Obstructive Sleep Apnea Syndrome as a Potential Cause of Nocturia in Younger Adults. Urology.

[4] Senaratna C.V., Perret J.L., Lodge C.J., Lowe A.J., Campbell B.E., Matheson M.C., Hamilton G.S., Dharmage S.C. (2017). Prevalence of obstructive sleep apnea in the general population: A systematic review. Sleep Med. Rev..

[5] Di Bello F., Napolitano L., Abate M., Collà Ruvolo C., Morra S., Califano G., Capece M., Creta M., Scandurra C., Muzii B. (2023). Nocturia and obstructive sleep apnea syndrome: A systematic review. Sleep Med. Rev..

[6] Umlauf M.G., Chasens E.R., Greevy R.A., Arnold J., Burgio K.L., Pillion D.J. (2004). Obstructive sleep apnea, nocturia and polyuria in older adults. Sleep.

[7] Kemmer H. (2009). The relationship between sleep apnea and overactive bladder. Curr. Urol. Rep..

[8] Yilmaz Z., Voyvoda B., Şirinocak P.B. (2018). Overactive bladder syndrome and bladder wall thickness in patients with obstructive sleep apnea syndrome. Int. Braz. J. Urol..

[9] Kemmer H., Mathes A.M., Dilk O., Gröschel A., Grass C., Stöckle M. (2009). Obstructive sleep apnea syndrome is associated with overactive bladder and urgency incontinence in men. Sleep.

[10] Dinç M.E., Avinçsal M.Ö., Balcı M.B.C., Özdemir C. (2018). Effect of Continuous Positive Airway Pressure on Overactive Bladder Symptoms in Patients with Obstructive Sleep Apnea Syndrome. Turk. Arch. Otorhinolaryngol..

[11] Ipekci T., Cetintas G., Celik O., Ekin R.G., Sarac S., Tunckiran A., IIbey Y.O. (2016). Continuous positive airway pressure therapy is associated with improvement in overactive bladder symptoms in women with obstructive sleep apnea syndrome. Cent. Eur. J. Urol..

[12] Deger M., Surmelioglu O., Kuleci S., Akdogan N., Dagkiran M., Tanrisever I., Yucel S.P., Izol V. (2021). The effect of treatment of obstructive sleep apnea syndrome on overactive bladder symptoms. Rev. Assoc. Med. Bras..

[13] Heinzer R., Vat S., Marques-Vidal P., Marti-Soler H., Andries D., Tobback N., Mooser V., Preisig M., Malhotra A., Waeber G. (2015). Prevalence of sleep-disordered breathing in the general population: The HypnoLaus study. Lancet Respir. Med..

[14] Danforth K.N., Townsend M.K., Lifford K., Curhan G.C., Resnick N.M., Grodstein F. (2006). Risk factors for urinary incontinence among middle-aged women. Am. J. Obstet. Gynecol..

[15] Tsujimura A., Takao T., Miyagawa Y., Yamamoto K., Fukuhara S., Nakayama J., Kiuchi H., Suganuma N., Nakamura T., Kumano-Go T. (2010). Urgency is an independent factor for sleep disturbance in men with obstructive sleep apnea. Urology.

[16] Grimby A., Milsom I., Molander U., Wiklund I., Ekelund P. (1993). The influence of urinary incontinence on the quality of life of elderly women. Age Ageing.

[17] Clerget A., Kanbar A., Abdessater M. (2020). Troubles urinaires et génito-sexuels dans le syndrome d’apnée obstructive du sommeil: Revue de la littérature. Prog. Urol..

[18] Chen J., Liu Z., Yang L., Zhou J., Ma K., Peng Z., Dong Q. (2024). Sleep-related disorders and lower urinary tract symptoms in middle-aged and elderly males: A cross-sectional study based on NHANES 2005–2008. Sleep Breath.

[19] Tuncer M., Yazici O., Kafkasli A., Sabuncu K., Salepci B., Narter F., Gungor G.A., Yucetas U. (2017). Critical evaluation of the overactive bladder and urgency urinary incontinence association with obstructive sleep apnea syndrome in a relatively young adult male population. Neurourol. Urodyn..

[20] Myer E.N.B., Long A., Cooper C., Fashokun T., Abernethy M., Chen C.C.G. (2020). Prevalence of Screening High Risk of Obstructive Sleep Apnea Among Urogynecology Patients. Female Pelvic Med. Reconstr. Surg..

[21] Zhou J., Xia S., Li T., Liu R. (2020). Association between obstructive sleep apnea syndrome and nocturia: A meta-analysis. Sleep Breath.

[22] Bostan O.C., Akcan B., Saydam C.D., Tekin M., Dascı O., Balcan B. (2021). Impact of Gender on Symptoms and Comorbidities in Obstructive Sleep Apnea. Eurasian J. Med..

[23] Maeda T., Fukunaga K., Nagata H., Haraguchi M., Kikuchi E., Miyajima A., Yamasawa W., Shirahama R., Narita M., Betsuyaku T. (2016). Obstructive sleep apnea syndrome should be considered as a cause of nocturia in younger patients without other voiding symptoms. Can. Urol. Assoc. J..

[24] Kang S.-H., Yoon I.-Y., Lee S.D., Kim J.-W. (2012). The impact of sleep apnoea syndrome on nocturia according to age in men. BJU Int..

[25] Rathmann W., Bongaerts B., Carius H.-J., Kruppert S., Kostev K. (2018). Basic characteristics and representativeness of the German Disease Analyzer database. Int. J. Clin. Pharmacol. Ther..

[26] Kaur N., Vetter C., Konrad M., Kostev K. (2024). Investigation of the Association between Sleep Disorders with Subsequent Depression in Children and Adolescents-A Retrospective Cohort Study with 62,796 Patients. Children.

[27] Loosen S., Krieg S., Krieg A., Leyh C., Luedde T., Vetter C., Kostev K., Roderburg C. (2023). Are sleep disorders associated with the risk of gastrointestinal cancer?-A case-control study. J. Cancer Res. Clin. Oncol..

[28] Jacob L., Kostev K. (2020). Urinary and fecal incontinence in stroke survivors followed in general practice: A retrospective cohort study. Ann. Phys. Rehabil. Med..

[29] Quan H., Sundararajan V., Halfon P., Fong A., Burnand B., Luthi J.-C., Saunders L.D., Beck C.A., Feasby T.E., Ghali W.A. (2005). Coding algorithms for defining comorbidities in ICD-9-CM and ICD-10 administrative data. Med. Care.

